# Nanoscale perfluorocarbon expediates bone fracture healing through selectively activating osteoblastic differentiation and functions

**DOI:** 10.1186/s12951-020-00641-2

**Published:** 2020-06-03

**Authors:** Shunhao Wang, Jiahuang Qiu, Anyi Guo, Ruanzhong Ren, Wei He, Sijin Liu, Yajun Liu

**Affiliations:** 1 State Key Laboratory of Environmental Chemistry and Ecotoxicology, Research Center for Eco-Environmental Sciences, Chinese Academy of Sciences, 8 Shuangqing Road, Haidian District, Beijing, 100085 China; 2 University of Chinese Academy of Sciences, Beijing, 100049 China; 3 Beijing Jishuitan Hospital, The 4th Clinical Hospital of Peking University Health Science Center, No. 31 East Street, Xinjiekou, Xicheng District, Beijing, 100035 China

**Keywords:** Bone fracture, Healing, Nano-PFC, Osteoblast, Differentiation

## Abstract

**Background and rationale:**

Fracture incidence increases with ageing and other contingencies. However, the strategy of accelerating fracture repair in clinical therapeutics remain a huge challenge due to its complexity and a long-lasting period. The emergence of nano-based drug delivery systems provides a highly efficient, targeted and controllable drug release at the diseased site. Thus far, fairly limited studies have been carried out using nanomedicines for the bone repair applications. Perfluorocarbon (PFC), FDA-approved clinical drug, is received increasing attention in nanomedicine due to its favorable chemical and biologic inertness, great biocompatibility, high oxygen affinity and serum-resistant capability. In the premise, the purpose of the current study is to prepare nano-sized PFC materials and to evaluate their advisable effects on promoting bone fracture repair.

**Results:**

Our data unveiled that nano-PFC significantly enhanced the fracture repair in the rabbit model with radial fractures, as evidenced by increased soft callus formation, collagen synthesis and accumulation of beneficial cytokines (e.g., vascular endothelial growth factor (VEGF), matrix metalloprotein 9 (MMP-9) and osteocalcin). Mechanistic studies unraveled that nano-PFC functioned to target osteoblasts by stimulating their differentiation and activities in bone formation, leading to accelerated bone remodeling in the fractured zones. Otherwise, osteoclasts were not affected upon nano-PFC treatment, ruling out the potential target of nano-PFC on osteoclasts and their progenitors.

**Conclusions:**

These results suggest that nano-PFC provides a potential perspective for selectively targeting osteoblast cell and facilitating callus generation. This study opens up a new avenue for nano-PFC as a promising agent in therapeutics to shorten healing time in treating bone fracture.
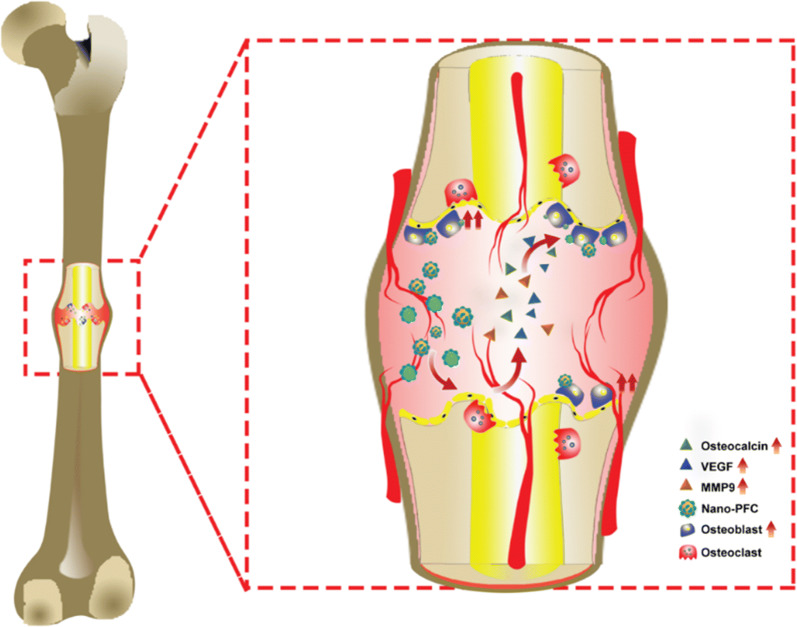

## Background

Fracture is a most common bone morbidity, due to population ageing and increasing traumas caused by industrial activities, transports and physical exercise [1]. The fracture healing has been proposed to be a complex biological process, including inflammatory reaction, cartilaginous callus formation, bony callus formation and bone remodeling process [2]. Thereby, accelerating fracture healing is critical for clinical therapeutics, but the current strategies that are able to promote osteogenesis remain rather limited. Intriguingly, biological therapies can greatly revolutionize the situation faced by traditional stargates, such as nonunion or delayed fracture healing after screws fixation, effective improving the clinical outcome. To date, the biological therapies (e.g., hormones, bone morphogenetic proteins and other growth factors) have been burgeoningly applied in therapeutics to enhance fracture repair [3]. However, these treatment strategies are often accompanied by many unfavorable off-target complications (e.g., infusion reaction, palpitations and immune impair) in addition to poor drug stability and high healthcare cost [4, 5]. Thus, additional edge-cutting, high efficacy and safe-treatment approaches are urgently warranted to improve fracture healing process.

The current composites or hybrid materials could not integrate well into the host tissue, and oftentimes result in foreign-body reaction, infection and possible extrusion of implanted materials. In this respect, nanotechnology provide a new tool to devise the structure of scaffold as well as to create drug delivery system with controllable release pattern, which has attracted widespread attention to date. Compared to traditional administration routes and methods, highly efficient nano-based drug delivery systems (NDDSs) achieve targeted drug delivery, high drug-loading capacity, improvement of drug solubility/stability and finetuned drug release in numerous biomedical indications. For even though the current studies on the bone repair applications dependent on nanomaterials and nanotechnology are fairly limited, burgeoning evidence hints the promising usage of nanodrugs in bone filed. For instance, a fracture-targeted nanoparticle delivery system for a GSK-3β inhibitor, a β-catenin agonist, was developed to enhance bone healing, showing excellent drug accumulation at the fracture sites with sustained release [4]. The agonist expedites fracture healing via activating Wnt/β-catenin signal and improving osteogenesis of osteoblast and mesenchymal stem cells, but eliciting no effect on osteoclasts. Such application of nanotechnology facilitated the targeted delivery of chemotherapeutics, and also enhanced the overall effect of drug in bone diseases and bone regeneration [6]. Nonetheless, since it is still in the infancy stage, there are still great challenges in developing NDDSs for bone fracture healing, such as insufficient drug-loading capacity, premature leakage and low targeting efficacy, which hinders the progression of clinical transformation [7, 8]. To this end, more desirable nanomedicines should be searched for the purpose of bone fracture healing treatment.

PFC, a clinically approved drug, is attracting increasing interest due to their chemical and biologic inertness, great biocompatibility, high oxygen affinity and serum-resistant capability [9, 10]. PFC could be effectively and readily eliminated through exhaled breath and reticuloendothelial system [11, 12]. Moreover, PFC-based research has also been verified to enhance the regeneration of soft tissue through elevated oxygen delivery [13, 14]. Importantly, PFC emulsion at the micro/nano size has been used in clinical practice for ultrasonography imaging, organ injury repair and emergency transfusion [15–17]. Recently, PFC emulsion at the nanoscale, here named nano-PFC, functioning as the oxygen shuttle, effectively relieved hypoxia microenvironment in the tumor associated with sensitized radiotherapy or photodynamic therapy [18–20], and also mitigated the hypoxia in the diabetic foot ulcer associated with enhanced wound healing, as reported in our study [21]. Therefore, as a new generation of NDDSs, nano-PFC offers enormous opportunities in enhancing bioavailability of drugs, prolonged half-life and targeted delivery for more potential applications.

The main objective of the current study was to verify the concept that nano-PFC could expediate bone fracture healing progression, which would be ascribed to a direct targeting of nano-PFC on bone healing microenvironment and bone cells. Surprisingly, we uncovered that nano-PFC administration increased the soft callus formation, collagen synthesis and the levels of beneficial cytokines, which were indispensably involved in bone healing. Mechanistic studies unveiled that nano-PFC targeted osteoblastic precursors to drive their differentiation and functions. Collectively, our results unearthed nano-PFC as promising nanomedicines in promoting bone fracture healing.

## Materials and methods

### Preparation and characterization of nano-PFC

Nano-PFC was prepared by modified micro-emulsion method [21]. In brief, 150 μL perfluoro-15-crown-5-ether (Fluorochem, UK) was added dropwise into 0.01 M phosphate-buffered saline (PBS, Solarbio, China) solution (4 mL) containing 1% Human serum albumin (HSA, Sigma-Aldrich, China). The mixed solution was vortexed for 10 s and emulsified with ultrasonic homogenizer (Scientz-1200E, China) for 200 s [20]. Then, nano-PFC materials were obtained through centrifugation (8000 r/min, 3 min), followed by washing. The size and morphology of nano-PFC were characterized by transmission electron microscope (TEM) (SU-8020, Hitachi, Japan) after negative staining using 1.5% phosphotungstic acid and Malvern zetasizer (NANO ZS, UK).

### Animal model of radius fracture

All animal experiments were approved by the Animal Ethics Committee of the Research Center for Eco-Environmental Sciences, Chinese Academy of Sciences. Male New Zealand White rabbits (5 months, 2.5–2.9 kg) were purchased from the Xinglong Experimental Animal Farm (Beijing, China). The rabbit radial fracture model was established after anesthesia with 0.1% pentobarbital sodium (40 mg/kg). The bone gap in radius was created using an orthopedic microelectric drill (Trauson Medical Instrument Co. Ltd, China). The diameter of the drill was 5 mm. Then, the wound was sutured after treatment with antibiotics [22]. The model rabbits were randomly divided into treatment group and control group. One week after operation, the rabbits were subjected to X-ray examination, and thereafter different treatments were carried out. The treatment group was intramuscularly injected with 5 μg/kg PFC weekly for the first 3 weeks, and the control group was injected with same weight saline. The intramuscular injection position was right within the fracture zones. Follow-up examinations were performed for 8 weeks.

### H&E and Masson staining

After 8-week treatment, the rabbits were sacrificed, and tissue specimens surrounding fracture locations were collected for further analyses. Afterwards, specimens were subsequently fixed in 10% PBS-buffered formaldehyde, followed by decalcification, embedding and sectioning. For H&E staining, the slides were incubated with hematoxylin (Solarbio, China) for 10 min and with Eosin (Solarbio, China) for 3 min, respectively. Thereafter, the slides were fixed with 70% ethanol for 20 s, 90% ethanol for 20 s, 100% ethanol for 60 s and xylene for 3 min. For Masson staining, the slides were stained with Weigert’s iron-hematoxylin (Solarbio, China) for 5 min, phosphomolybdic–phosphotungstic acid (Solarbio, China) for 45 s and a solution containing 1% orange G and 0.25% aniline blue (Solarbio, China) for 5 min. Next, these slides were rinsed with 1% acetate solution (Solarbio, China) and stained with 0.12% ponceau xylidine (Solarbio, China) for 20 min. After rinsing with 1% acetate solution, the slides were then incubated with 2.5% phosphotungstic acid (Solarbio, China) for 10 min, rinsed with 1% acetate solution, and dehydrated in ethanol and xylene. Histological images were collected through Pannoramic 250 Flash III (3DHISTECH Ltd, Budapest, Hungary), and were analyzed by CaseViewer 2.3 and Image J software (National Institutes of Health, USA) accordingly.

### Immunofluorescent staining

Fractured radius specimens were prepared for immunofluorescent staining according to the standard protocol [23]. Briefly, the deparaffinized tissue sections in citrate buffer were heated at 95 °C for 10 min for antigen retrieval, followed by blocking for 1 h with 10% normal mouse serum. The primary antibodies were used against VEGF (dilution 1:50, Abcam, USA), MMP-9 (dilution 1:100, Abcam, USA) and osteocalcin (10 μg/mL, Abcam, USA) at 4 °C overnight. Afterwards, the sections were incubated with the goat anti-mouse secondary antibody conjugated with FITC for 1 h at the room temperature. Finally, all slides were stained with 4′,6-diamidino-2-phenylindole (DAPI). Images of immunofluorescent staining were collected through Pannoramic 250 Flash III, and analyzed by CaseViewer 2.3 and Image J software, respectively.

### Cytotoxicity assay

Both RAW 264.7 and MG-63 cells were seeded into 96-well plates (8000 cells/well) overnight. Thereafter, nano-PFC at different concentrations was used to treat cells for 24 h. Then, the cell viability was immediately assessed by Cell Counting Kit-8 (CCK-8, Solarbio, 1000T, China) assay following a standard protocol with 3 independent experiments.

### Osteoblast differentiation in vitro

Human MG-63 cells were obtained from the American Type Culture Collection (ATCC), and cultured with Minimum Essential Medium (MEM, GIBCO) containing 10% fetal bovine serum (FBS, Hyclone) at 37 °C with 5% CO_2_. MG-63 cells were induced to differentiate under the conditioned medium with 50 μg/mL ascorbic acid (Sigma-Aldrich, China), 10 nM dexamethasone (R&D Systems, USA) and 20 nM β-glycerophosphate (R&D Systems, USA) for 7 days. To determine osteoblast maturation, cells after treatment with different concentrations of nano-PFC were stained with alkaline phosphatase (ALP), following the instructions provided by the manufacturer (Nanjing jiancheng bioengineering institute, China). The ALP staining in osteoblasts was quantified by the Image J software.

### Osteoclast differentiation in vitro

Mouse RAW 264.7 cells were also obtained from the ATCC and cultured with Dulbecco’s Modified Eagle’s Medium (DMEM, GIBCO), supplemented with 10% FBS (Hyclone) and 100 U/mL penicillin/streptomycin (Gibco). RAW 264.7 cells were induced towards mature osteoclasts by macrophage colony-stimulating factor (M-CSF, R&D Systems, USA) and receptor activator of nuclear factor kappa B ligand (RANKL, R&D Systems, USA) for 7 days, as described [24]. To determine the effects of nano-PFC on RAW 264.7 cell differentiation, cells after treatment with different concentrations of nano-PFC were stained with tartrate resistant acid phosphatase (TRAP), following the instructions provided by the manufacturer (Solarbio, China).

### RT-qPCR analysis

Total RNA was isolated from cells with Trizol reagent (Invitrogen, USA). The RNA concentration was measured with a nanodrop ND-1000 instrument (Thermo Fisher Scientific, USA). Total RNA (in 6 μg) was reversely transcribed into cDNA with M-MLV reverse transcriptase (Promega, USA). The expression levels of target genes were examined with the standard SYBR green qPCR system on a CFX96 real-time instrument (Bio-Rad Inc., USA), as previously described [25]. Primers are listed in Additional file 1: Table S1. Here, β-actin was used as a loading control for normalization.

### Statistical analysis

Statistical analyses were carried out using the independent t-test and one-way ANOVA with the SPSS Statistics 17.0 software. All data are presented as mean ± standard error. Statistical significance is defined as P < 0.05 and P < 0.001.

## Results and discussion

### Characterization of nano-PFC materials

**Fig. 1:**
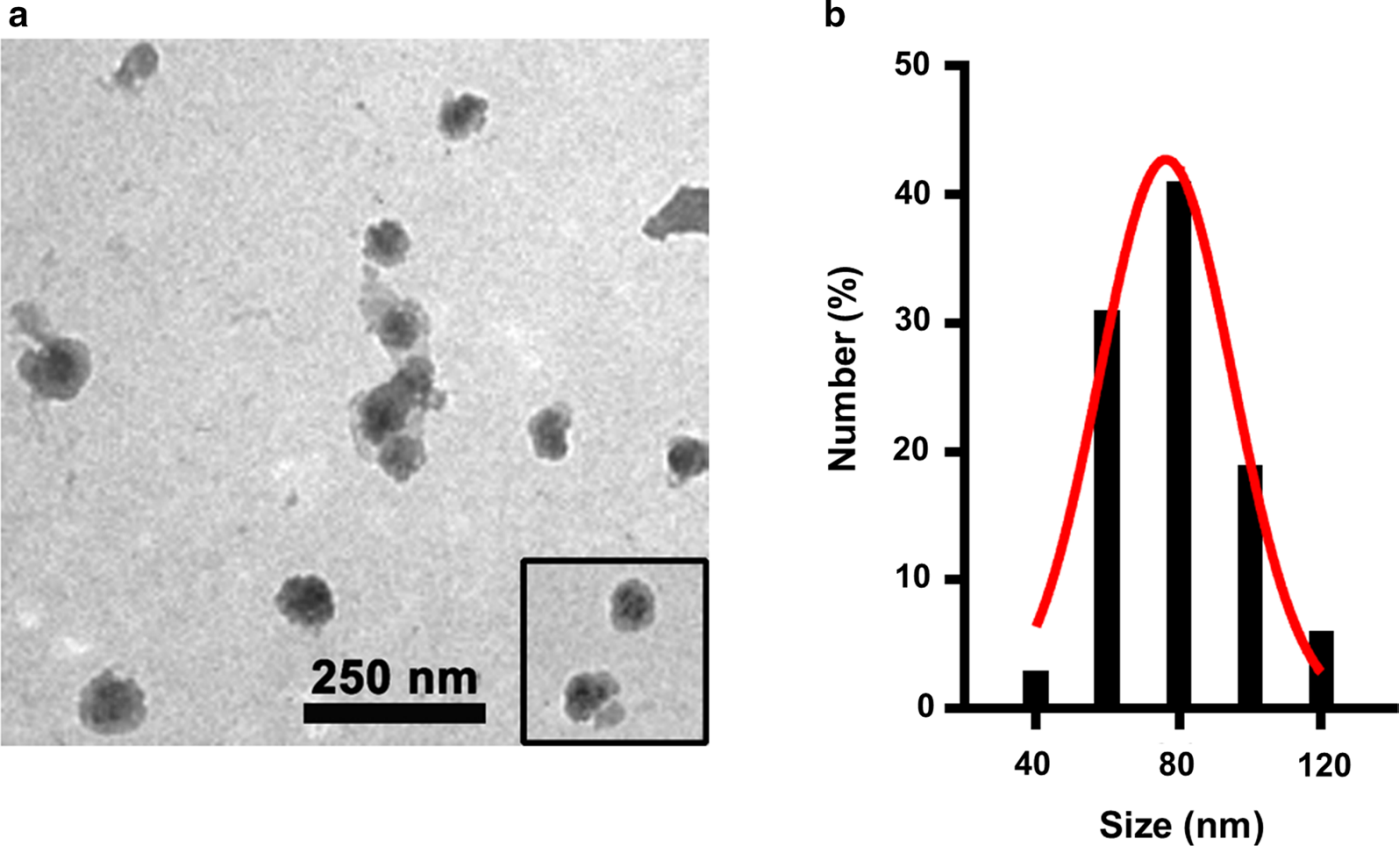
Characterization of nano-PFC. **a** A representative TEM image of HSA-stabilized nano-PFC with an insert showing their microstructure. **b** The size distribution profile of nano-PFC based on the TEM analysis with a Gaussian fit curve in the histogram

HSA-stabilized nano-emulsion PFC materials, abbreviated as nano-PFC, were prepared by micro-emulsion method under ultrasonication. As the diameter of bone sinusoids is roughly 80–100 nm, we deliberately fabricated the size of our nano-PFC to be around 80 nm. As characterized by TEM analysis in Fig. 1a, our nano-PFC particles displayed a uniform size distribution with an average diameter of approximately 80 nm (Fig. 1b). We also measured the size distribution profile of nano-PFC by DLS, as shown in Additional file 1: Figure S1. This hydrodynamic size was calculated to be about 100 nm, which was slightly larger than the results determined by TEM, which should be ascribed to the formation of the hydrodynamic shell, as demonstrated previously [26, 27]. The real size of nanoemulsion droplets can be measured unbiasedly by TEM and DLS, which together offer a more detailed insight into droplet size distribution. Together our nano-PFC realized a suitable size for extravasation towards bone microenvironment.

### Nano-PFC accelerated bone fracture healing in a rabbit model

**Fig. 2:**
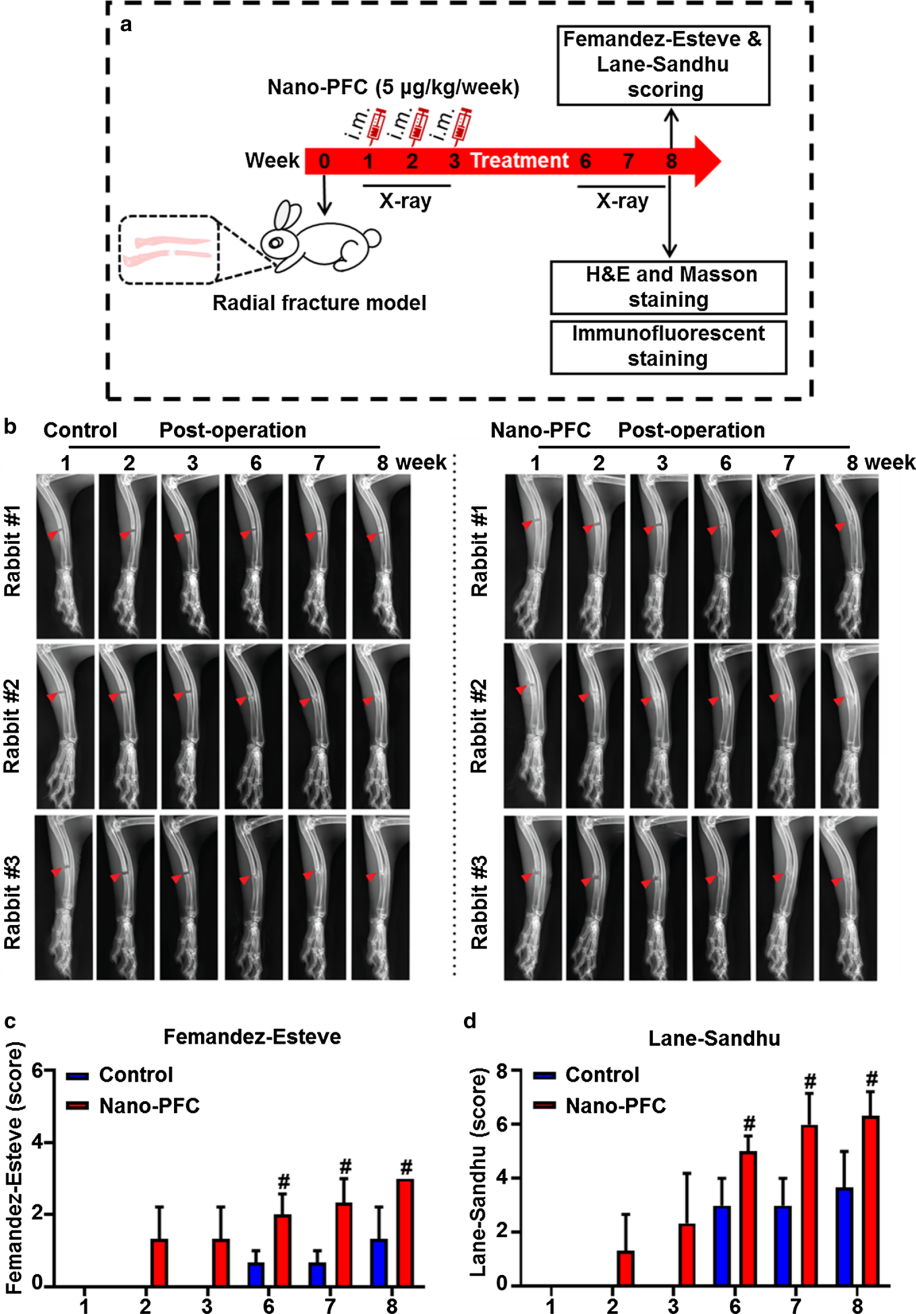
Nano-PFC functions to accelerate bone fracture healing. **a** A flow chart showing the overall experimental procedure. **b** The radial radiographs of representative animals in each group showing the healing progression over time (n = 3). Red arrowheads point at the fracture regions. **c** Femandez-Esteve and **d** Lane-Sandhu scores for the quantitative determination of bone healing progression. ^#^P < 0.001, compared to the control group at the according time points

To interrogate the promoting effects of nano-PFC on bone fracture healing, we first established a model using rabbits (Fig. 2a), as established in previous reports [28]. The rabbit model provides a more accurate system to study both phenotype changes and mechanisms, as these purposes could be readily reached in the mouse model that is limited by operational difficulty and insufficient specimens [29]. As shown in Fig. 2b, the X-ray images manifested that radius fracture was successfully created in our rabbits, where even gaps (~ 5 mm in width) were defined in radii. Since nano-PFC could be metabolized through bone sinusoids, animals were therefore locally administrated with nano-PFC once a week for 3 weeks (Fig. 2a). As shown in Fig. 2b, as the treatment progressed, significant calluses were gradually generated in the osteotomized bones over time in all animals. Strikingly, a remarkable difference was found between nano-PFC-treated rabbits and untreated control (Fig. 2b). Compared to untreated rabbits, the callus formation was greatly enhanced in treated animals, as reflected by the radial radiographs (Fig. 2b), suggesting reinforced bone healing in the osteotomized zones upon nano-PFC treatment. Quantitative evaluation, through determining the Femandez-Esteve and Lane-Sandhu scores, as established previously [30], showed rapid initiation of bone formation in nano-PFC treated animals within 3 weeks post treatment, in that considerable Femandez-Esteve and Lane-Sandhu scores were observed in treated group on 2 and 3 weeks after administration in comparison to no score in untreated control (Fig. 2c, d). Moreover, consecutively enhanced bone healing was found in the fractured zones over the time course from 6 to 8 weeks after nano-PFC administration, compared to control group (P < 0.05). Of note, nearly absolute fusion was observed in truncated bone ends after 8-week treatment in nano-PFC-treated animals, as reflected by the X-ray images (Fig. 2b).

**Fig. 3:**
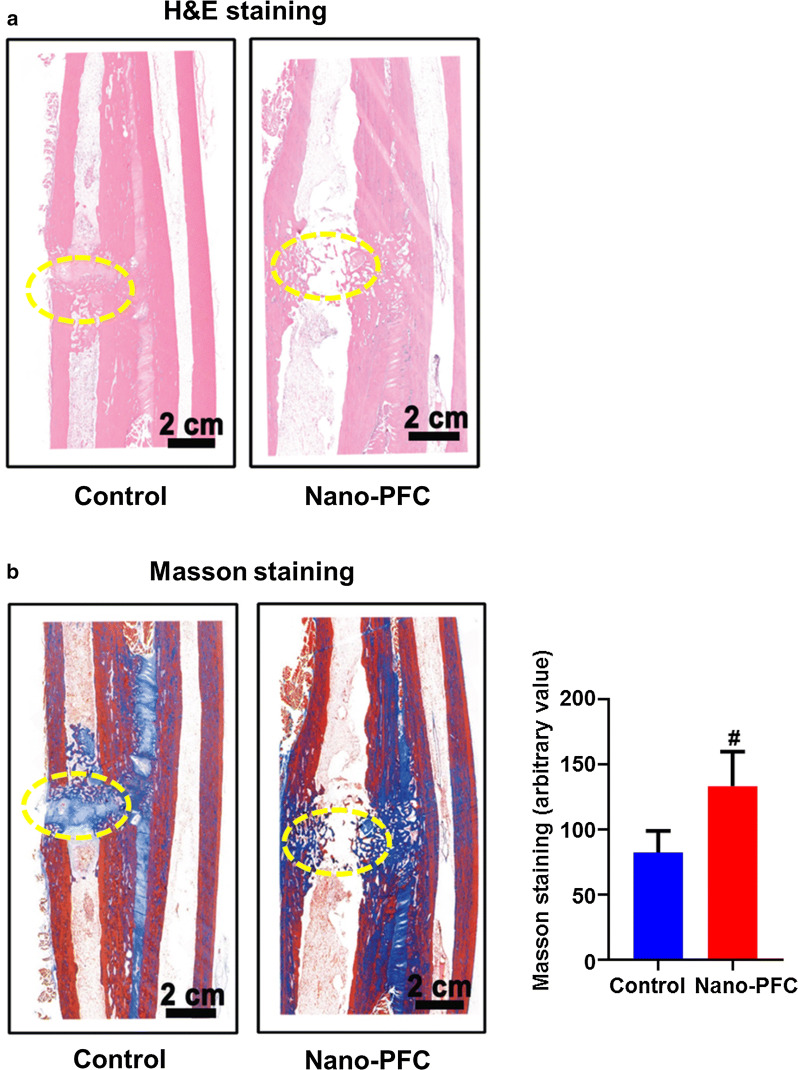
H&E and Masson staining of fracture zones after nano-PFC treatment. Radius were harvested from fracture regions from rabbits 8 weeks post operation. Representative images of **a** H&E staining and **b** Masson staining for each group. Circles indicate the visualization of fractured zones. The intensity of Masson staining was quantified for each group (n = 3). The scale bar is 2 cm

To corroborate the accelerated bone healing induced by nano-PFC, we further probed into the fracture regions through histological examination when animals were sacrificed after 8-week treatment. As shown in Fig. 3a, the H&E staining results showed massive localization of bone callus into truncated areas with complete filling of the gaps in nano-PFC-treated radii, in contrast to mild growth of bone callus into truncated sites with obvious crevices left between two broken ends in untreated control. These morphological differences signified the important contribution of nano-PFC to improving bone callus formation and growth into broken microenvironments. To substantiate this contribution of nano-PFC, the main component of bone callus, type I and type Ш collagen [31], was assessed through Masson staining (Fig. 3b). Our results displayed marked intrusion of collagen into fractured sites with dark blue color for treated animals; however, only mild collagen accumulation with light blue staining was found in untreated control (Fig. 3b). Quantitative analysis of the total regions of Masson staining determined by Image J software recognized more than twofold elevation of collagen intensity for treated rabbits relative to untreated ones (Fig. 3b, the right panel, P < 0.001). Noteworthily, nano-PFC-treated specimens showed more active bone tissue remodeling within the medullary canal with significant formation of trabeculae in treatment group, compared to untreated group (Fig. 3b). Consistent with the results from the radiographs (Fig. 2), these observations revealed that that nano-PFC significantly accelerated the bone repair and tissue remodeling in the rabbits with radial fractures.

### Increased bone formation activities in fractured zones in response to nano-PFC

**Fig. 4:**
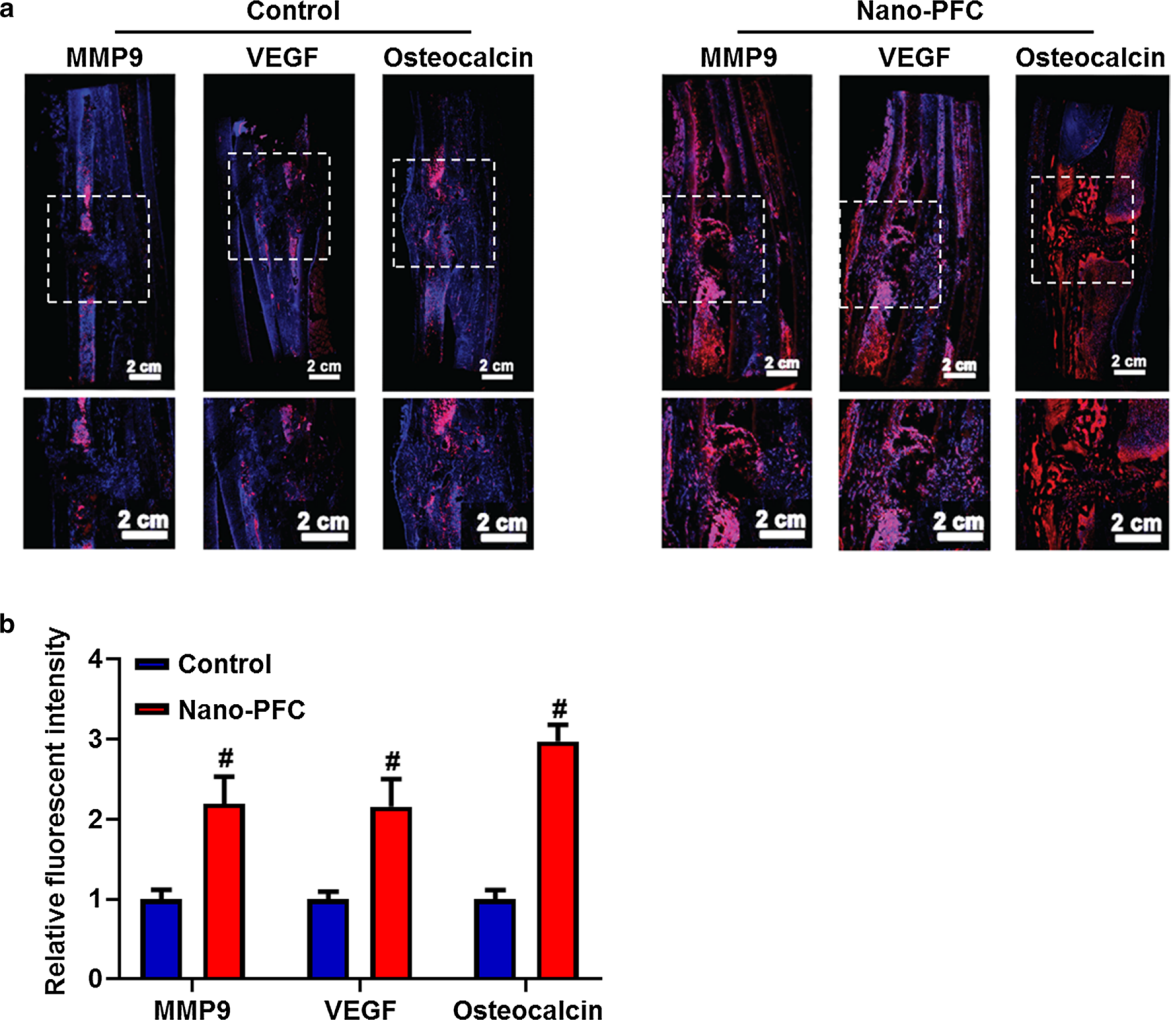
Immunofluorescent analysis of fracture zones after nano-PFC treatment for 8 weeks. **a** Representative immunofluorescent images of specimens with staining against MMP-9, VEGF and osteocalcin (in red). Meanwhile, nuclei are counter stained with DAPI (in blue). The scale bar is 2 cm. **b** Comparison of relative fluorescent intensity (n = 3). *P < 0.05, compared to the control group

In fact, a few studies have documented that nanomaterials (such as silica nanoparticles [32], gold nanoparticles [33] and nano tantalum implants [34]) could promote osteoblast differentiation and mineralization irrespective of exogenous growth factors. Inspired by the above findings on enhanced bone healing responding to nano-PFC, we intended to investigate the molecular events reflective of bone formation activities. Matrix metalloproteins (MMPs), in particular MMP-9, is closely implicated in the modulation of bone formation through finetuning the intricate balance between osteoblastic and osteoclastic activities [35]. MMP-9 regulates the bioavailability and bioactivity of transforming growth factor-β (TGF-β), RANKL and parathyroid hormone related protein (PTHrP), especially in response to altered conditions of bone homeostasis [36, 37]. Moreover, numerous studies also evidence MMP-9 as a critical regulator for the activation of the cascade of various MMPs and resultantly bone formation due to its regulation on the osteoblastic and osteoclastic activities [38]. To determine the levels of MMP-9 in repaired zones, immunofluorescent staining was performed. As shown in Fig. 4a, largely increased fluorescence (in red) was observed in the specimens from nano-PFC-treated animals in comparison to untreated control. Quantified data found more than twofold increase of fluorescent intensity in nano-PFC treated specimens, compared to untreated control (Fig. 4b).

Moreover, vascular injuries coupled to fracture lead to an ischemic environment [39, 40]. Whereby, insufficient blood supply seriously hinders the healing of the fracture. The hypoxic environment at the site of fracture, created by ischemia, would provoke cell death, delay chondrocyte and osteoblast differentiation, and therefore block fracture healing [41–43]. Based on previous reports [44, 45], VEGF is secreted by osteoblasts, chondrocytes endothelial progenitor cells (EPCs) and mesenchymal progenitors, and VEGF has been demonstrated to play a crucial role in the progress of fracture healing through promoting the invasion of vessels, functioning to alleviate the hypoxic microenvironment [46], and improving biogenesis of vascularized osseous tissue [47, 48]. More importantly, several research groups demonstrated that the highest expression levels of osteoblast-derived VEGF were found at the late differentiation stage [49, 50]. Moreover, MMP-9-mediated matrix degradation actually contributes to triggering the release of VEGF from the cartilage matrix, and consequently enhances the vascular invasion into growing bone microenvironment [51]. In analogy to the change of MMP-9, the level of VEGF was also largely induced by nano-PFC compared to untreated control, as characterized by immunofluorescent staining (Fig. 4a). Approximately twofold induction of VEGF level was found in nano-PFC-treated specimens, compared to untreated specimens, as reflected by the quantitative data (Fig. 4b, P < 0.05). Based on above findings, we speculated that the nano-PFC with high oxygen affinity, upon injection into the fracture site, might allow more oxygen to be delivered and stored at the site of the fracture, and thereby promote osteoblastic differentiation and functions through improving the hypoxic environment [42]. To this end, the elevated oxygen would further accelerate osteoblast differentiation, and then increase the levels of osteoblast activity-related proteins (e.g., MMP-9 and VEGF), resulting in promoted fracture healing. In a word, the oxygen supply, benefiting from nano-PFC with high oxygen affinity, played an indispensable role in the process of fracture healing.

Further, we attempted to look into the priming state of osteoblasts, which could more realistically identify the bone formation activity. As a most representative surrogate, osteocalcin, as a noncollagenous protein in bone, is produced by osteoblasts and is defined to be a marker in recognizing bone formation due to its role in mineralization and calcium ion homeostasis [52, 53]. Additionally, osteocalcin is also necessary in bridging calluses [54]. Figure 4a exhibits massive osteocalcin accumulation in the healing regions from nano-PFC-administrated animals, as evidenced by considerable immunofluorescent staining, in contrast to slight staining in untreated animals. Furthermore, quantitative analysis unveiled the increase of fluorescent intensity by nearly 3 times (Fig. 4b). To this end, it would be concluded that nano-PFC greatly elevated the priming state of osteoblasts by reinforcing their activities and functions.

### Nano-PFC mechanistically promotes the osteoblastic differentiation and functions

**Fig. 5:**
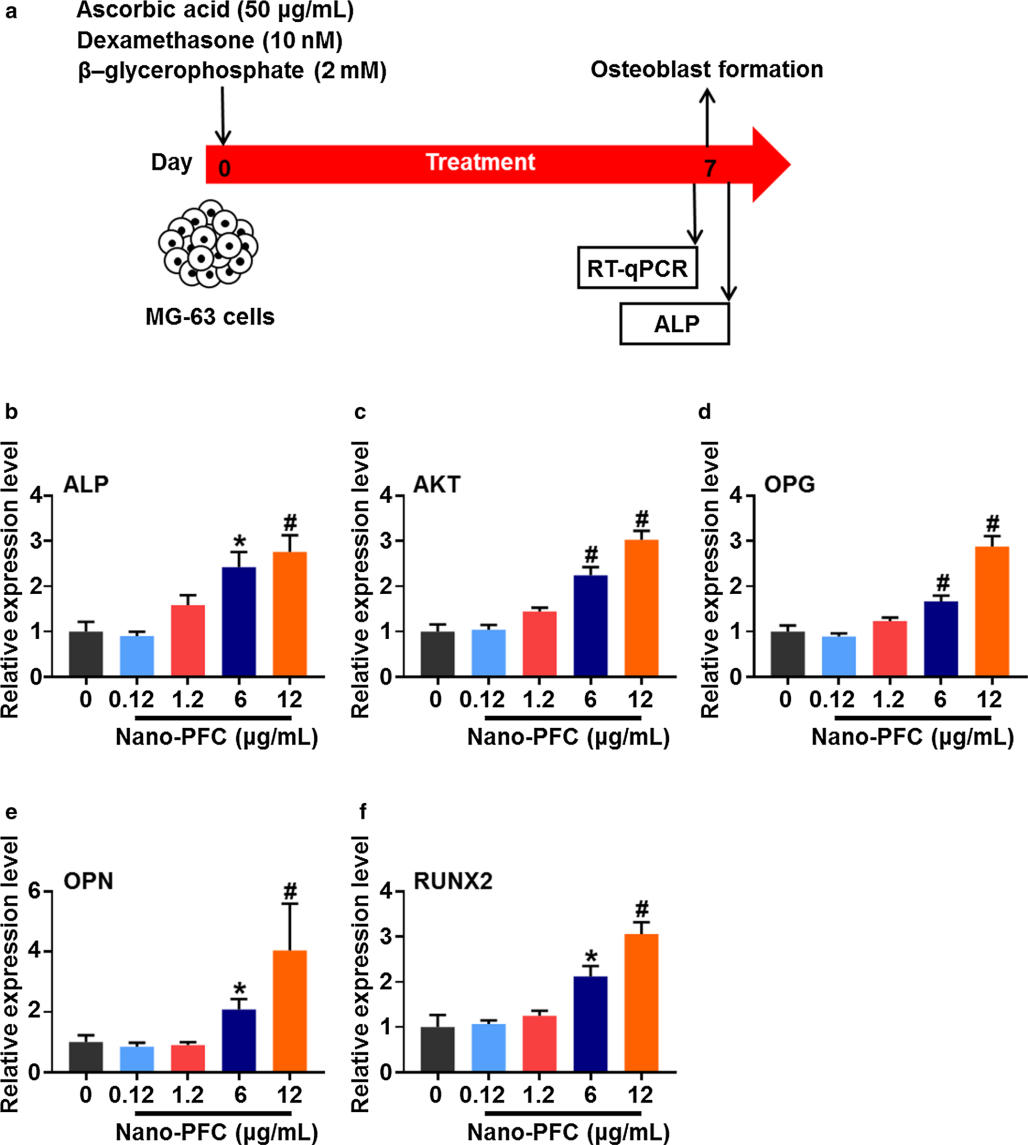
Nano-PFC accelerates the differentiation of MG-63 cell-derived osteoblasts. **a** Schematic illustration of MG-63 cell-induced differentiation under conditioned medium. **b**–**f** Evaluation of MG-63 cell differentiation towards osteoblasts using representative marker genes including ALP, AKT, OPG, OPN and RUNX2 after 7 days induction with or without nano-PFC treatment at various concentrations (n = 6). *P < 0.01 and ^#^P < 0.001, compared with the control group

**Fig. 6:**
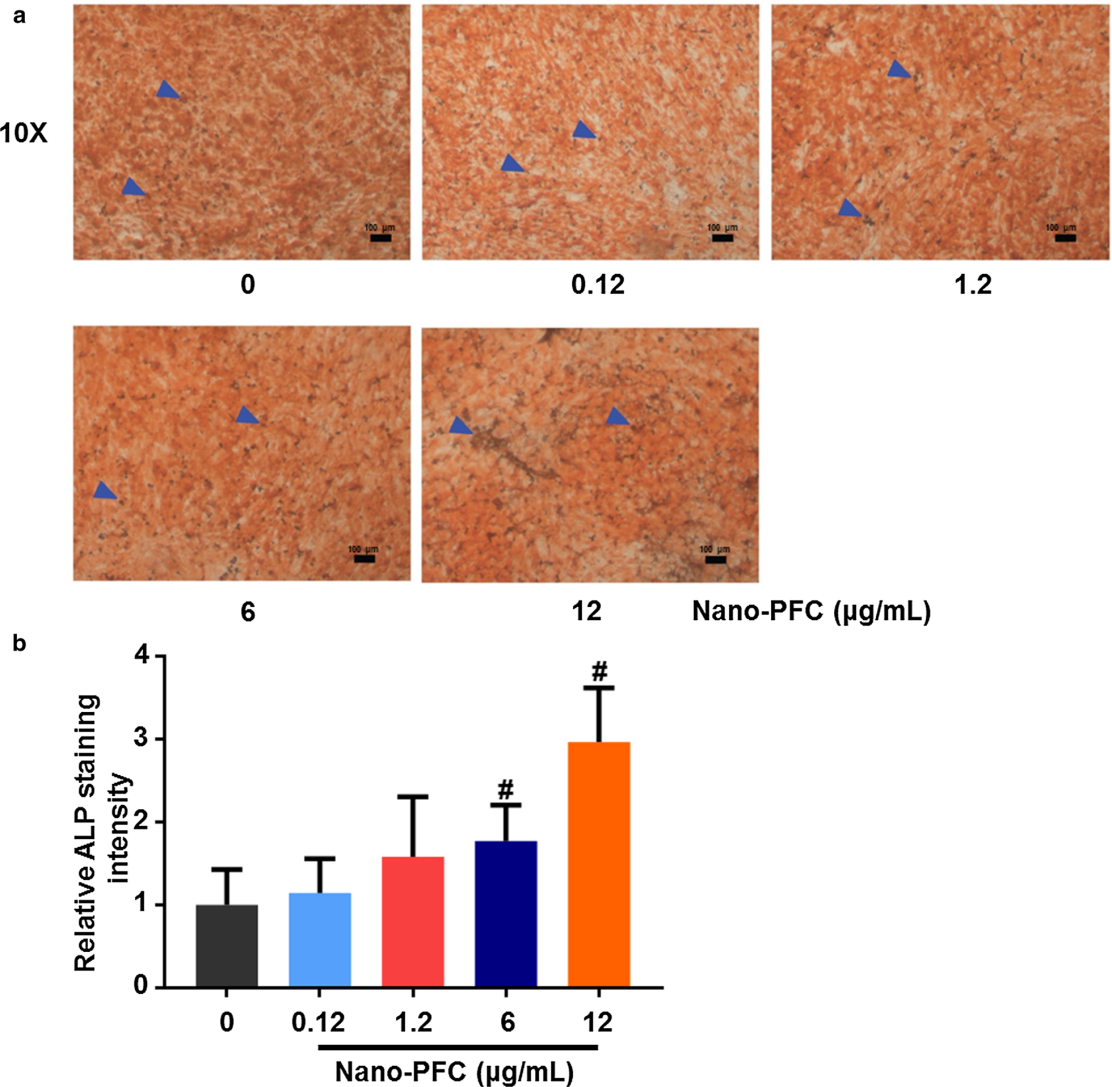
ALP determination of MG-63 cell-derived osteoblasts with or without nano-PFC treatment. **a** Representative images of ALP staining after nano-PFC treatment at different concentrations for 7 days. Blue arrowheads indicate positive ALP staining in mature osteoblasts. The scale bar is 100 μm. **b** Quantified data of ALP staining. ^#^P < 0.001, compared with the control group

Our above results collectively unearthed nano-PFC-induced strong effects on bone formation, which could be ascribed to altered activities of both osteoblasts and osteoclasts. These encouraging results incited us to figure out the target cells and according molecular mechanisms. For this purpose, we studied the likely influences of nano-PFC on osteoblasts and osteoclasts. Bone formation is essential for fracture healing, in which osteoblasts drive this process through secreting mineralization-related factors (e.g., ALP) on the surface of bone tissue [55]. Thus, to explore this likelihood in osteoblasts, a commonly used cell line, MG-63, representative of osteoblast progenitor, was employed for the study of osteoblastic differentiation and functions upon nano-PFC in vitro [56]. To initiate osteoblastic differentiation, MG-63 cells were induced in the presence of ascorbic acid, dexamethasone and β-glycerophosphate (as delineated in Fig. 5a), which mimic bone healing microenvironments in vitro [57]. To find out the desirable non-toxic concentrations of nano-PFC in MG-63 cells, we first screened the cell viability of MG-63 cells. As shown in Additional file 1: Figure S2, nano-PFC did not incur significant toxicity towards MG-63 cells even up to 12 μg/mL, as determined by the CCK-8 method, indicating marked biocompatibility and biosafety of nano-PFC in MG-63 cells. Upon induction of MG-63 cells in conditioned medium, successful differentiation of MG-63 cells was identified, as evidenced by the signification induction of gene expression for a number of osteoblast biomarkers including ALP, bone gamma-carboxyglutamate protein (BGLAP), osteoprotegerin (OPG), osteopontin (OPN) and runt-related transcription factor 2 (RUNX2) determined by RT-qPCR (Additional file 1: Figure S3, P < 0.001). ALP, a homodimeric protein with phosphorylating feature, is a typical marker of osteoblast differentiation, which is expressed in 1–2 week of osteogenic stage and functions to implement a stimulating effect on tissue mineralization [58]. Phosphoinositide 3-kinase and protein kinase B signaling pathway (PI3K/AKT) can enhance normal skeleton formation through regulating osteoblast differentiation and homeostasis [59]. BGLAP and OPN represent specific non-collagen bone matrix proteins that are synthesized and secreted by mature osteoblasts [60, 61]. Among them, OPG is a glycoprotein that is primarily synthesized by osteoblasts, and it acts to inhibit osteoclastic differentiation and bone resorption activity through binding to RANKL [62]. Moreover, osteoblast progenitors are induced to differentiate into mature osteoblasts under the driving force of transcription factors, in particular RUNX2, followed by extracellular matrix deposition and mineralization, which is indispensable for the integration of new bone components into the fracture site [63]. Of note, oxygen availability at the fracture site is a key regulator of osteoblast differentiation [64]. The elevated oxygen can significantly increase RUNX2 expression, which further promotes the local mass of VEGF and thus vascular invasion for the fracture repair and new tissue growth [65, 66]. RUNX2 is a crucial transcription factor in promoting osteoblast differentiation by enhancing the expression of important osteoblastic genes including ALP, AKT, BGLAP and OPN [67]. In analogy to the in vivo findings (Fig. 5), upon nano-PFC induction, an overall dose-dependent increase of these genes were observed (P < 0.05), and the greatest induction was found in cells upon the highest concentration at 12 μg/mL, with two to fourfold increase relative to untreated cells (Fig. 5b–f, P < 0.001). Greater activities of osteoblast, together with high expression of differential biomarker, indicate that nano-PFC expedited fracture healing by improving osteogenesis of osteoblast. To confirm these results, ALP staining was carried out at the end of induction for 7 days. Figure 6a displays positive staining (in brown) of ALP for all cells cultured in conditioned medium; however, even darker color was observed in nano-PFC-treated cells, particularly at higher concentrations. Quantification showed approximately two and threefold increase of ALP staining upon nano-PFC at 6 and 12 μg/mL, respectively (Fig. 6b). Together, these results unveiled the outstanding capability of nano-PFC to induce osteoblast differentiation and bone formation functions.

### Nano-PFC elicited no effect on osteoclastic differentiation and activity

**Fig. 7:**
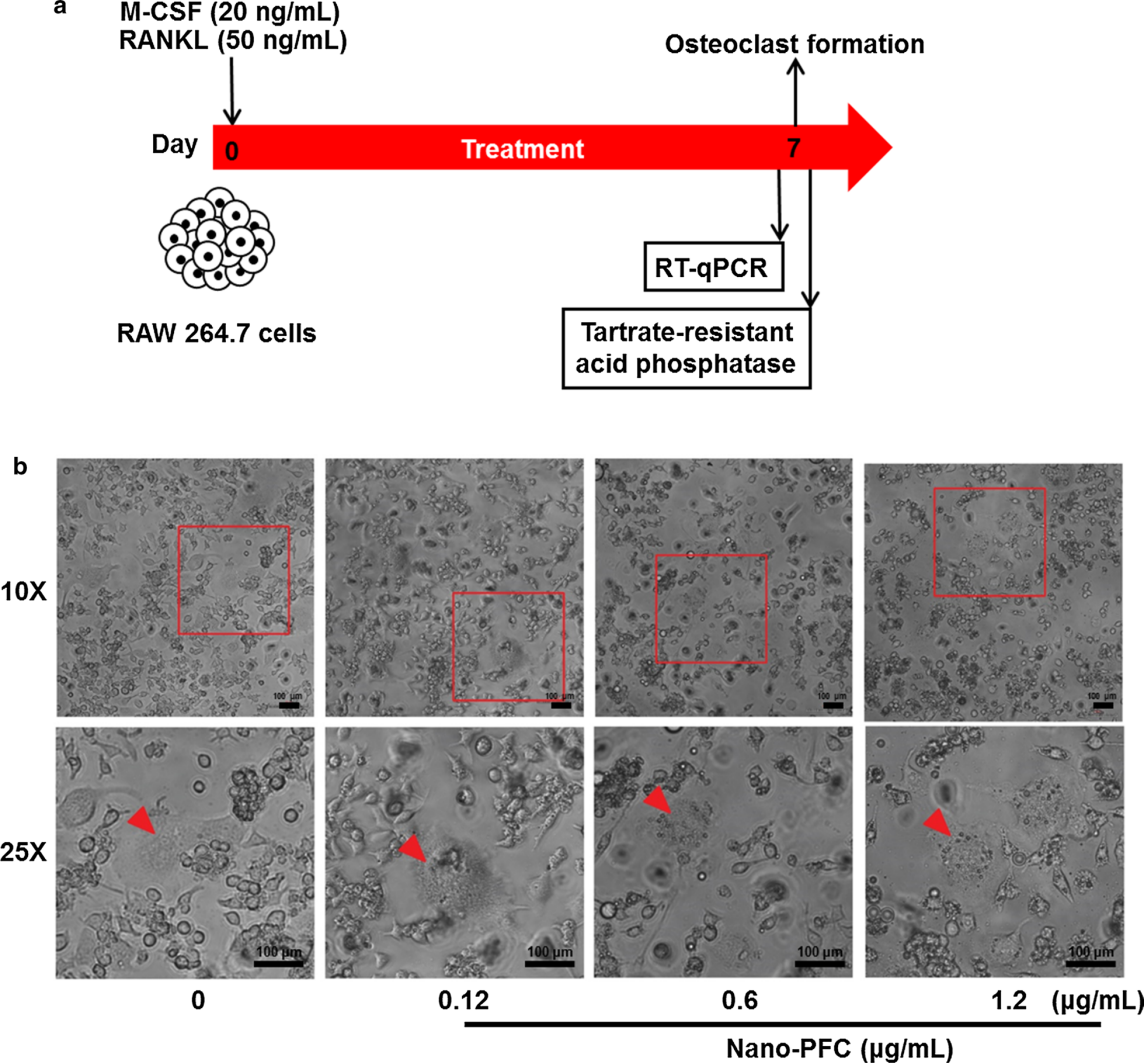
Evaluation of RAW 264.7 cell differentiation towards osteoclasts. **a** Schematic illustration of RAW 264.7 cell induction towards osteoclasts under conditioned medium. **b** Morphological characterization of RAW 264.7 cell differentiation towards osteoclasts upon nano-PFC at various concentrations for 7 days. The scale bar is 100 μm. Red arrowheads indicate mature osteoclasts

Next, we also endeavored to interpret the possible impact of nano-PFC on osteoclastic differentiation and bone resorption. In the process of bone remodeling, osteoclasts indispensably account for bone resorption by cleaving bone matrix by secreting H^+^ and enzymes, which is also critical for fracture healing [68]. Without orchestrated osteoclastic activities, in other words, either aggressively overactivated osteoclasts or impaired osteoclast would undermine concerned bone reconstruction program [61, 69]. Under this context, balanced interplay between osteoblasts and osteoclasts is of great importance in modulating normal bone homeostasis and remodeling. Similar to the studies on osteoblasts, a widely recognized osteoclast cell line, RAW 264.7, was used for the determination of osteoclast differentiation under conditioned medium with M-CSF and RANKL (as depicted in Fig. 7a) [70]. Consistent with previous studies [71], RAW 264.7 cells were successfully induced into mature osteoclasts under conditioned culture medium, as reflected by the remarkable induction of representative osteoclastic markers including carbonic anhydrase II (CA-II) [72], nuclear factor of activated T-cells (NFATC) [73] and TRAP [74], as determined by RT-qPCR (Additional file 1: Figure S4, P < 0.001). Moreover, morphological changes also verified the maturation of RAW 264.7 cells into osteoclasts, such as multinucleated osteoclast-like cells with larger size, as reported [75], as shown in Fig. 7b.

**Fig. 8:**
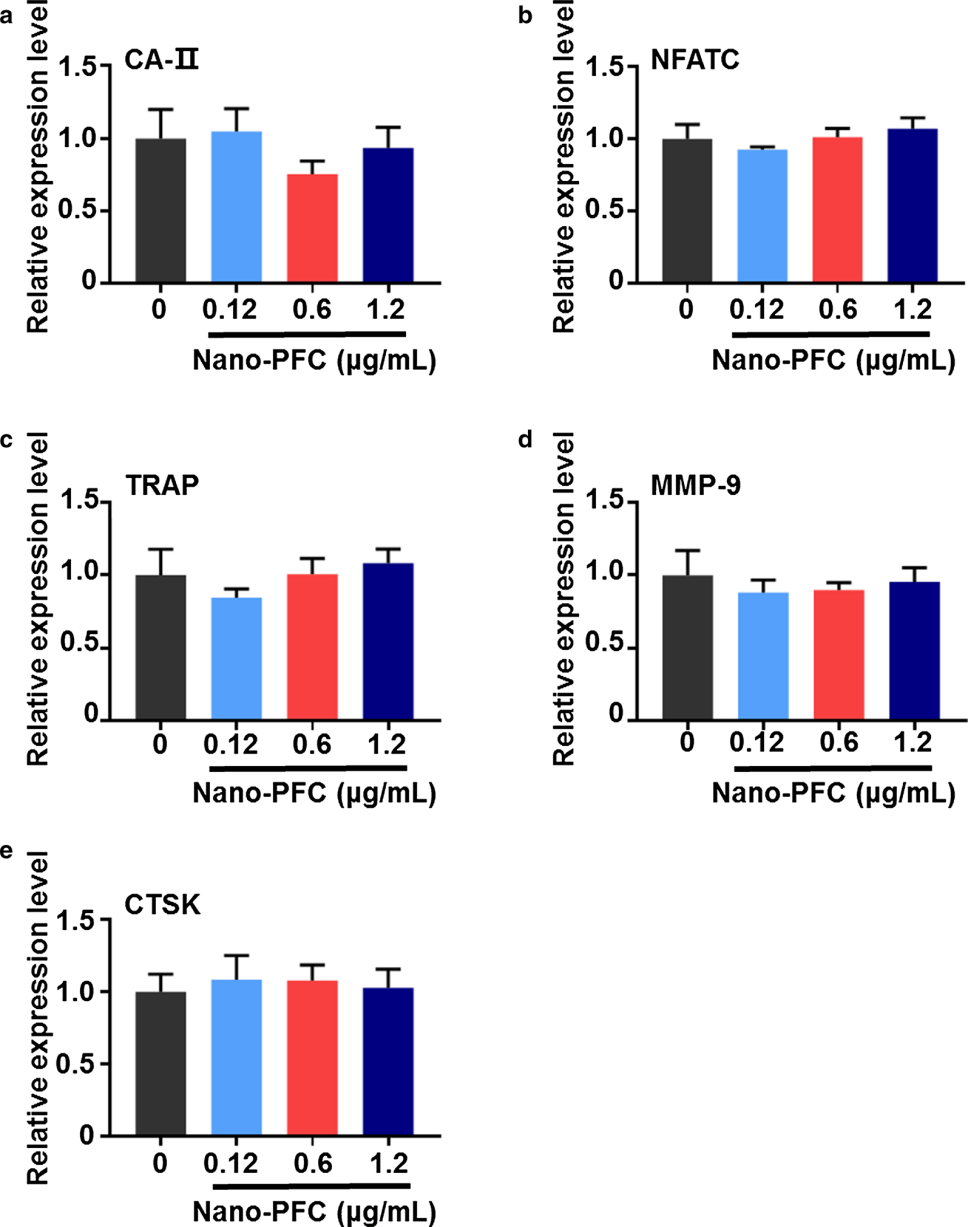
Determination of RAW 264.7 cell differentiation towards osteoclasts using representative marker genes. **a** CA-II, **b** NFATC, **c** TRAP, **d** MMP-9 and **e** CTSK were determined using RT-qPCR in induced cells after 7-day treatment with nano-PFC at various concentrations (n = 6)

**Fig. 9:**
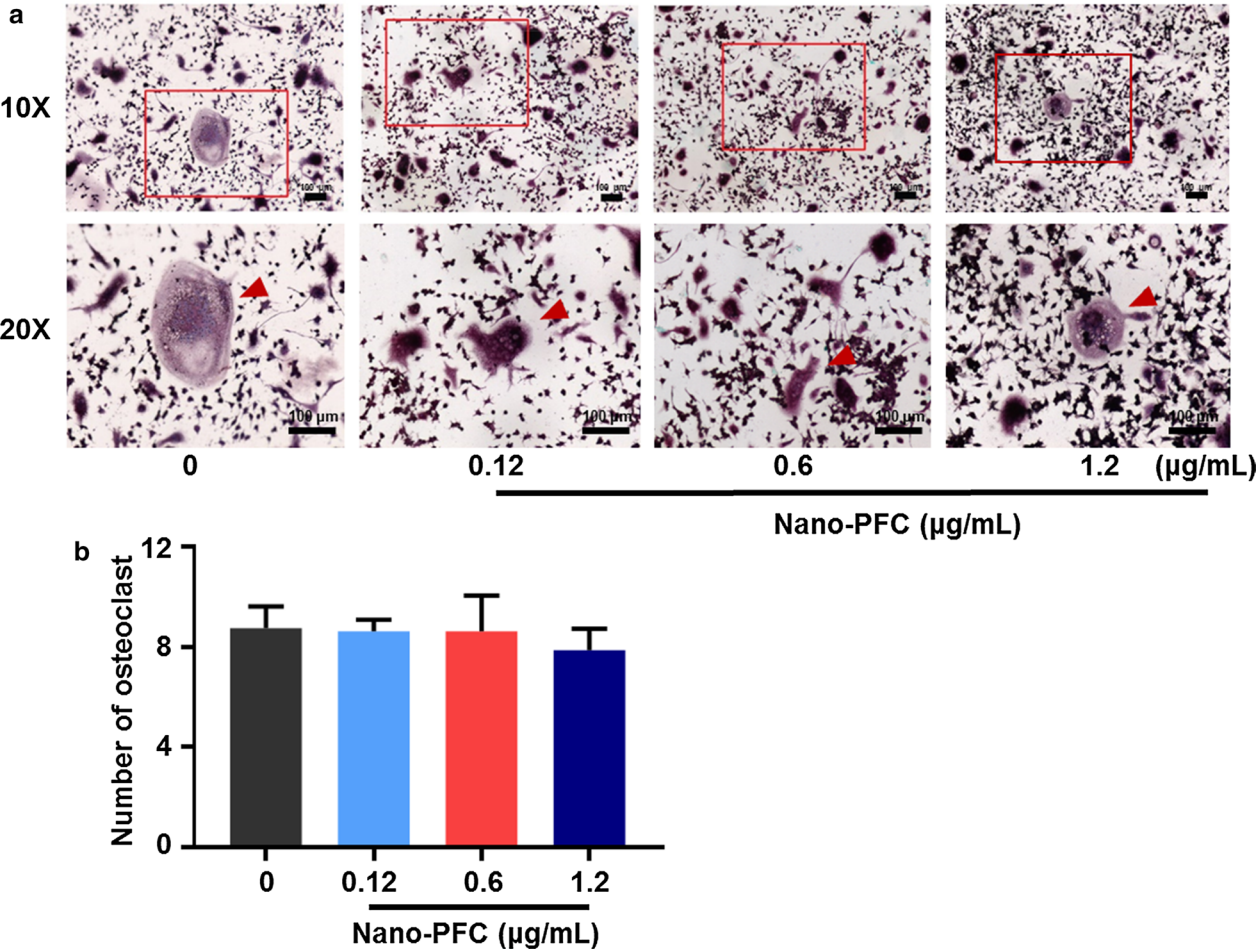
Examination of TRAP activity. **a** Representative images of TARP staining of nano-PFC-treated cells at different concentrations for 7 days in comparison to untreated cells. The scale bar is 100 μm. Red arrowheads indicate mature osteoclasts. **b** Quantitative analysis of TARP staining showing the number of mature osteoclasts. The number of mature osteoclasts were counted in each random field (n = 8)

Afterwards, the cytotoxicity of nano-PFC was assayed in RAW 264.7 cells through the method of CCK-8. As shown in Additional file 1: Figure S5, very different from MG-63 cells, cyto-compatibility was demonstrated in RAW 264.7 cells at relatively low concentrations. Under this setting, low-dose exposure was performed in RAW 264.7 cells. We further researched osteoclast differentiation in RAW 264.7 cells after Nano-PFC treatment through the evaluation of a number of osteoclastic hallmarks by RT-qPCR. As shown in Fig. 8, no significant induction of these osteoclastic hallmarks was observed in RAW 264.7 cells upon nano-PFC at various concentrations, including CA-II, NFATC, TRAP, MMP-9 and cathepsin K (CTSK). To substantiate these data, TRAP staining was further determined in nano-PFC-treated cells in comparison to untreated cells. As shown in Fig. 9, very clear TRAP^+^ multinucleated cells were observed under induced conditions; however, no difference was found for the number of TRAP^+^ multinucleated cells upon nano-PFC treatment, compared to untreated cells (Fig. 9b). Consistently, phase-contrast microscopy unraveled pronounced multinucleated cells upon induction, but no significant variation was observed in response to nano-PFC treatment at different concentrations (Fig. 9a). Collectively, our data suggested that nano-PFC did not target osteoclast precursors, but rather acted to promote osteoblastic differentiation and activation during the process of fracture healing.

## Conclusions

**Fig. 10:**
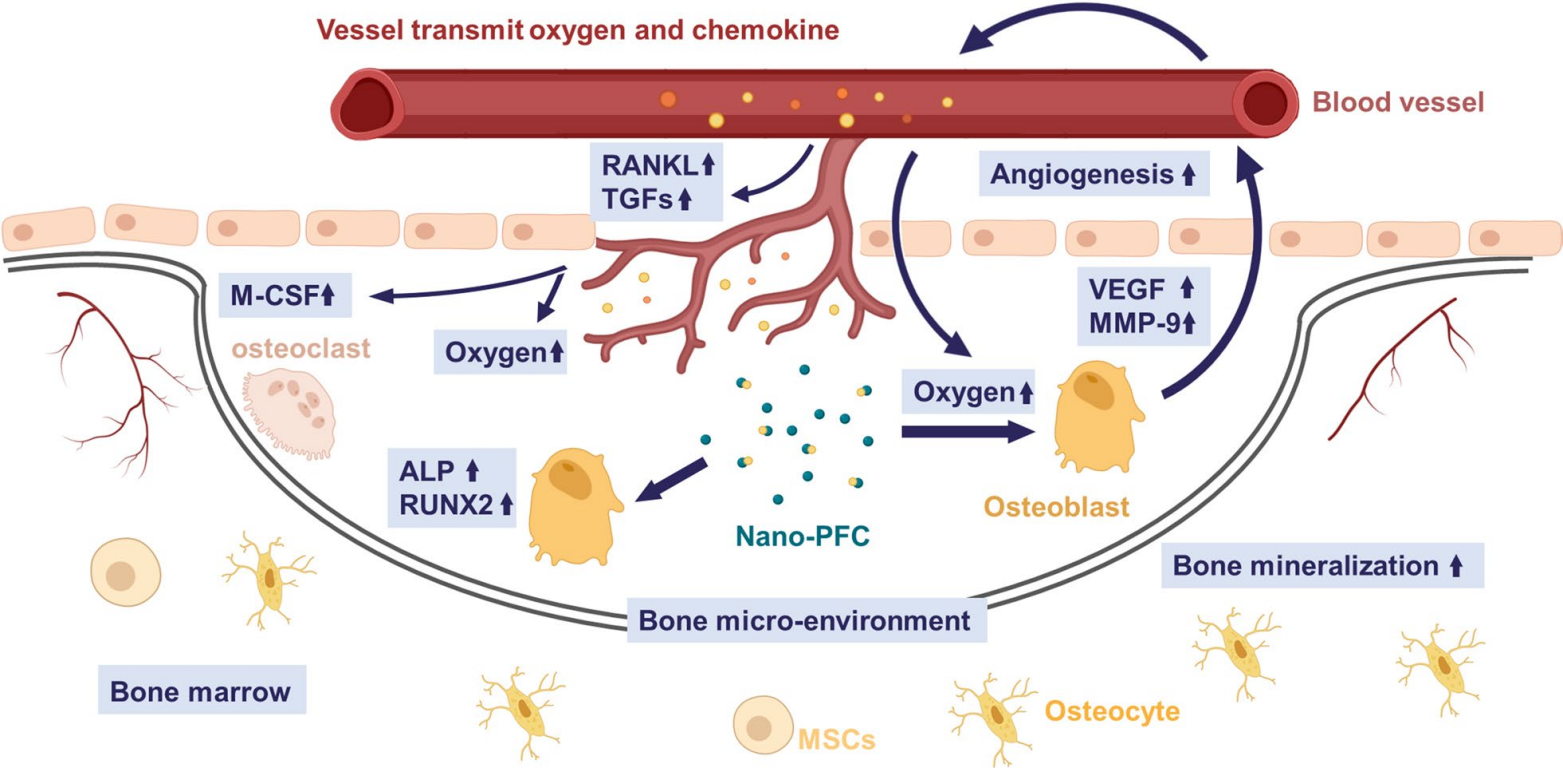
A proposed schematic diagram deciphering nano-PFC-promoted bone fracture healing

Enhanced bone healing is a great challenge in orthopedics, coupled with unsolved questions on the strategies of targeting drugs towards bone microenvironment or osteoblasts with less adverse side effects and clinical complications. In this study, PFC, a clinically approved drug, was nanosized to nanomedicines in addressing this challenge. Our nano-PFC nanomedicines were fabricated to fit the right size of bone sinusoids in order for perfect effectively localization within bone fracture sites. Consistent data uncovered that nano-PFC greatly accelerated bone fracture healing in a rabbit model. Mechanistic studies unraveled that nano-PFC functioned to greatly enhance the local concentrations of VEGF, MMP-9 and osteocalcin within the fracture microenvironment, and these factors acted together to stimulate bone repair and remodeling (Fig. 10). Moreover, our results uncovered that nano-PFC targeted osteoblasts to induce their differentiation and functions that are necessary for new bone formation. An overall proposed schematic delineating nano-PFC-promoted bone fracture healing is illustrated in Fig. 10. Together, this study unearthed a remarkable potential of nano-PFC in efficiently facilitating bone fracture healing progress.

## Supplementary information

**Additional file 1.** Additional figures and table.

## Data Availability

All data and materials in this study are included in the published article and its additional file.

## Abbreviations

PFC: Perfluorocarbon
VEGF: Vascular endothelial growth factor
MMP-9: Matrix metalloprotein 9
NDDSs: Nano-based drug delivery systems
PBS: Phosphate-buffered saline
TEM: Transmission electron microscope
DAPI: 4′,6-Diamidino-2-phenylindole
ATCC: American Type Culture Collection
HAS: Human serum albumin
MMPs: Matrix metalloproteins
TGF-β: Transforming growth factor-β
RANKL: Nuclear factor kappa B ligand
PTHrP: Parathyroid hormone related protein
EPCs: Endothelial progenitor cells
ALP: Alkaline phosphatase
CCK-8: Cell counting kit-8
BGLAP: Gamma-carboxyglutamate protein
OPG: Osteoprotegerin
OPN: Osteopontin
RUNX2: Runt-related transcription factor 2
M-CSF: Macrophage colony-stimulating factor
CA-II: Carbonic anhydrase II
NFATC: Nuclear factor of activated T-cells
TRAP: Tartrate resistant acid phosphatase
CTSK: Cathepsin K
