# Supplementary material for: Nanoscale perfluorocarbon expediates bone fracture healing through selectively activating osteoblastic differentiation and functions
Source: J Nanobiotechnology. 2020 Jun 3;18:84. doi: 10.1186/s12951-020-00641-2 (PMC7271395; doi:10.1186/s12951-020-00641-2)
Supplement: Supplementary file 1 — Additional file 1. Additional figures and table. [file 12951_2020_641_MOESM1_ESM.docx]

**Additional Figure Legend.**


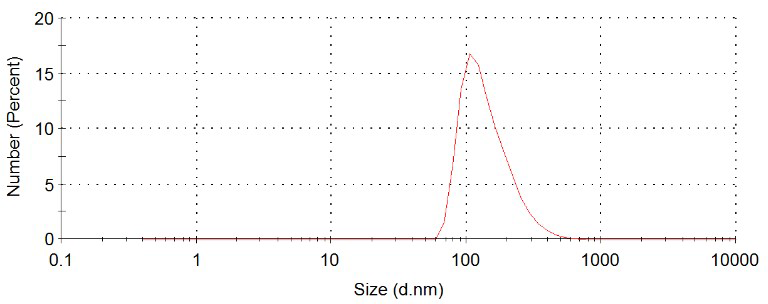


**Figure S1.** Size distribution of nano-PFC as measured by DLS.

**
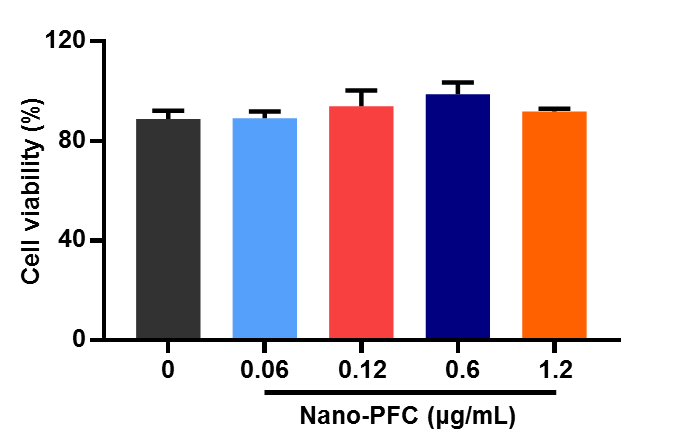
**

**Figure S2.** Cell viability was determined with the CCK-8 method in MG-63 cells upon nano-PFC treatment at different concentrations for 24 h (n=5).


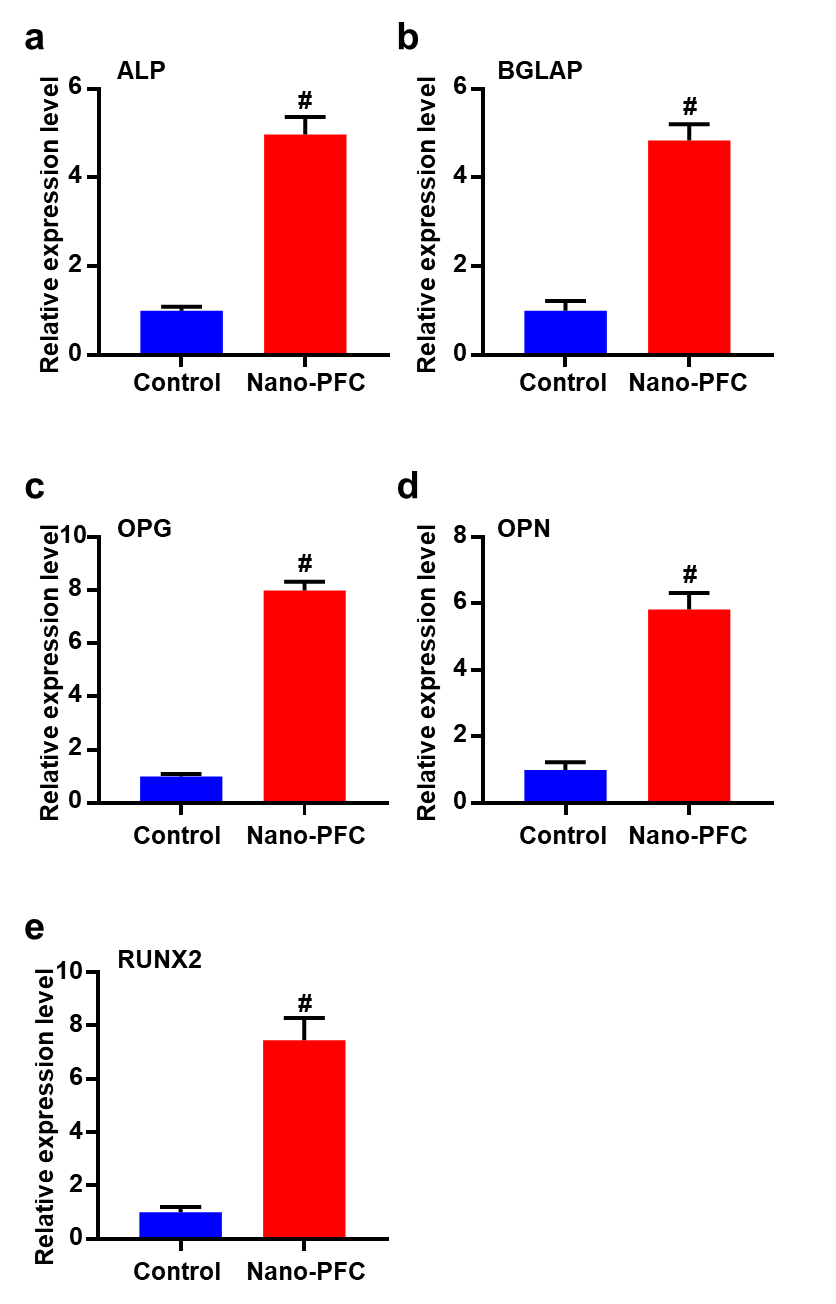


**Figure S3.** Expression levels of surrogate genes in MG-63 cells upon induction in conditioned medium by RT-qPCR (n=6). #: P<0.001, compared to uninduced cells.

| 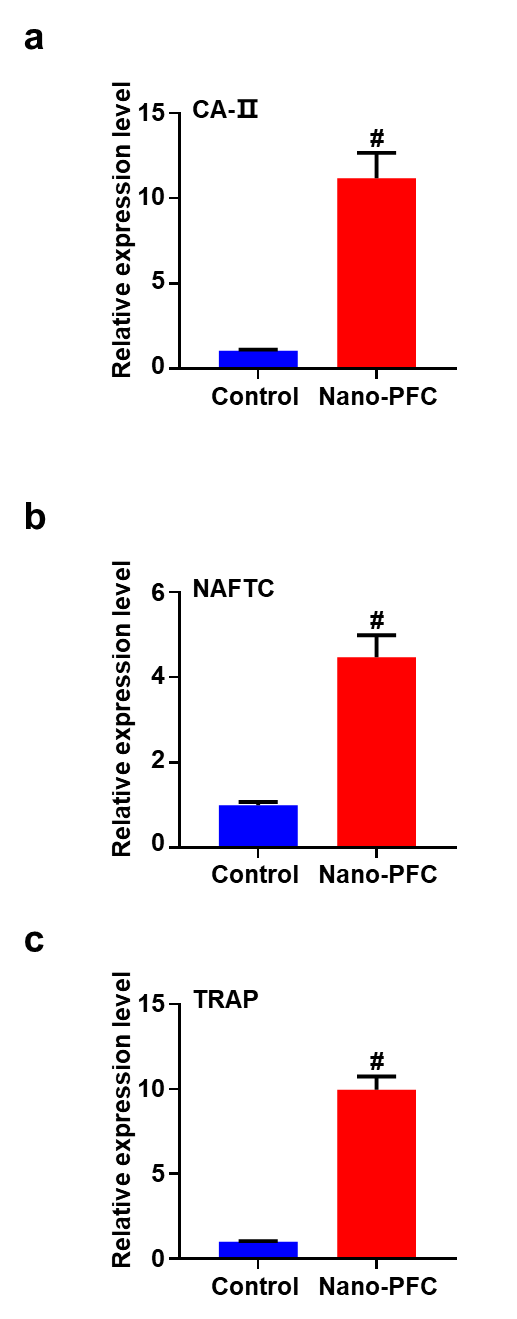  **Figure S4.** Differentiation evaluation of RAW 264.7 cell-derived osteoclasts through surrogate gene expression by RT-qPCR analysis. These representative marker genes include **a** CA-II, **b** NFATC and **c** TRAP (n=6). #: P<0.001, compared to the control group. |
| --- |


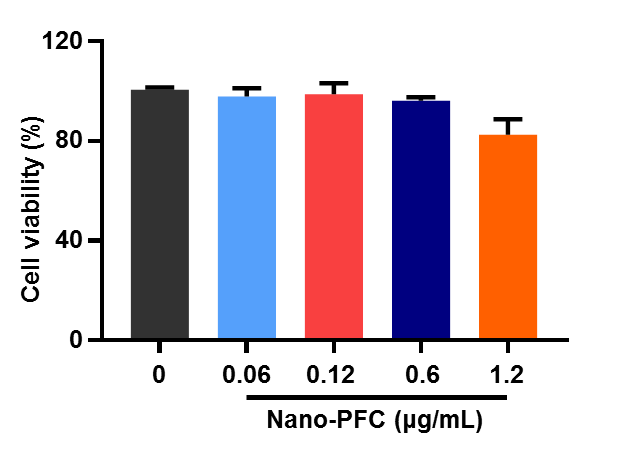


**Figure S5.** Cell viability was determined with the CCK-8 method in RAW 264.7 cells after nano-PFC treatment at different concentrations for 24 h (n=5).

**Table S1.** Primer sequences for RT-qPCR.

|  |
| --- |
